# 1.12 Å resolution crystal structure of the catalytic domain of the plasmid-mediated colistin resistance determinant MCR-2

**DOI:** 10.1107/S2053230X17009669

**Published:** 2017-07-26

**Authors:** Katie Coates, Timothy R. Walsh, James Spencer, Philip Hinchliffe

**Affiliations:** a School of Cellular and Molecular Medicine, University of Bristol, Bristol BS8 1TD, England; b Department of Medical Microbiology and Infectious Disease, Cardiff Institute of Infection and Immunity, UHW Main Building, Heath Park Hospital, Cardiff CF14 4XN, Wales

**Keywords:** MCR-1, antibiotic resistance, colistin, MCR-2, polymixin

## Abstract

MCR-2 confers resistance to colistin, a ‘last-line’ antibiotic against extensively resistant Gram-negative pathogens. It is a plasmid-encoded phosphoethanol­amine transferase that is closely related to MCR-1. To understand the diversity in the MCR family, the 1.12 Å resolution crystal structure of the catalytic domain of MCR-2 was determined. Variable amino acids are located distant from both the di-zinc active site and the membrane-proximal face. The exceptionally high resolution will provide an accurate starting model for further mechanistic studies.

## Abstract 2

The crystal structure of the plasmid-mediated colistin resistance determinant MCR-2 has been determined at 1.12 Å resolution. This high-resolution structure highlights the molecular diversity of clinically relevant MCR proteins and provides an accurate starting model for further mechanistic, and in particular computational, studies.

## 1. Introduction   

The polymyxin colistin is a key ‘last-resort’ antibiotic used to treat infections by multidrug-resistant Gram-negative pathogens (Biswas *et al.*, 2012 ▸; Karaiskos *et al.*, 2017 ▸). The positively charged cyclic peptide of colistin binds to the negatively charged lipid A headgroup, with the hydrophobic tail inserting into, and disrupting, the outer membrane (Clausell *et al.*, 2007 ▸; Wiese *et al.*, 2003 ▸). A key colistin resistance mechanism is the production of MCR-1 (Liu *et al.*, 2016 ▸), a plasmid-encoded phosphoethanolamine transferase that has disseminated worldwide. It is found in clinical strains of *Escherichia coli* and *Klebsiella pneumoniae* (Liu *et al.*, 2016 ▸), and in bacteria producing other resistance determinants, such as carbapenemases (Mediavilla *et al.*, 2016 ▸; Haenni *et al.*, 2016 ▸), which can result in essentially untreatable bacterial infections.

MCR-1 catalyses the transfer of positively charged phosphoethanolamine onto lipid A, which is subsequently incorporated into the outer membrane, reducing the net negative charge and preventing colistin binding (Hinchliffe *et al.*, 2017 ▸; Liu *et al.*, 2016 ▸). It is an integral, metal-dependent inner-membrane protein, with a large periplasmic domain containing the catalytic centre and the conserved Thr285 that is likely to act as the acceptor for the phosphoethanolamine group during the transfer reaction (Hinchliffe *et al.*, 2017 ▸). We recently described two crystal structures of the MCR-1 catalytic domain (MCR-1^CD^), revealing the presence of one (PDB entry 5lrn; MCR-1^5LRN^) or two (PDB entry 5lrm; MCR-1^5LRM^) zinc ions in the active site (Hinchliffe *et al.*, 2017 ▸), with the proposed catalytic Thr285 phosphorylated or not phosphorylated, respectively. Additional MCR-1^CD^ structures have been reported: two with phosphorylated Thr285 and crystallized from conditions with a nonphysiologically high zinc content [PDB entries 5gov (MCR-1^5GOV^; Hu *et al.*, 2016 ▸) and 5k4p (MCR-1^5K4P^; Stojanoski *et al.*, 2016 ▸)], and consequently containing additional zinc ions, and one with two active-site zinc ions and both phosphorylated and nonphosphorylated Thr285 (PDB entry 5grr; MCR-1^5GRR^; Ma *et al.*, 2016 ▸). More recently, the full-length, detergent-solubilized crystal structure of an MCR homologue (EptA; 36% sequence identity to MCR-2) was solved (Anandan *et al.*, 2017 ▸) with a single zinc ion, a nonphosphorylated Thr285 and a bound molecule of dodecyl maltoside (DDM) in the active site. This full-length structure confirmed the prediction (Hinchliffe *et al.*, 2017 ▸) that the active site is proximal to the membrane.

**Figure 1:**
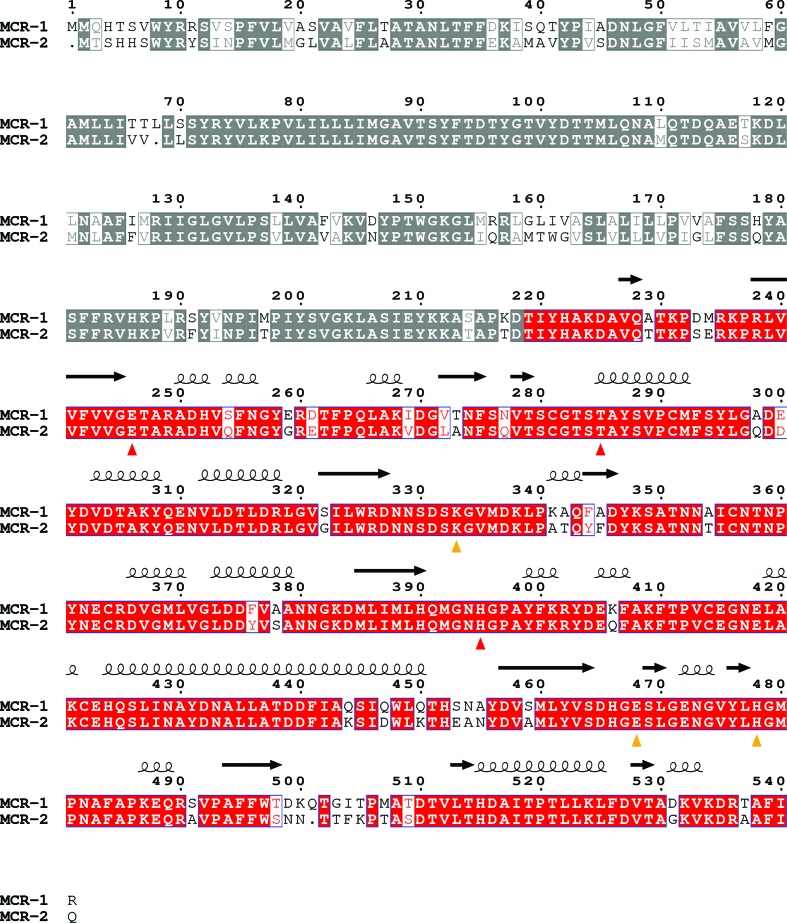
Sequence alignment of MCR-1 (541 residues) and MCR-2 (538 residues). Strictly conserved residues are boxed in white on a red background and highly conserved residues are boxed in red on a white background. The putative membrane domain is greyed out (residues 1–218). Secondary structure is indicated above based on the MCR-2^CD^ crystal structure. Residues where mutations reduce MCR-1 activity to basal levels are indicated by red triangles, and residues that are important to MCR-1 activity (*i.e.* mutation significantly reduces but does not abolish activity) are indicated by yellow triangles.

Two genes closely related to *mcr-1* have also been identified. Firstly, *mcr1.2*, containing a Gln3Leu substitution, was found in a *K. pneumoniae* strain (Di Pilato *et al.*, 2016 ▸). Secondly, *mcr-2* was detected in colistin-resistant *E. coli* isolated from porcine and bovine samples, with a higher prevalence than *mcr-1* in the porcine samples (Xavier *et al.*, 2016 ▸). *mcr-2* is harboured on a plasmid (IncX4) with a high transfer frequency that appears to lack a fitness cost to the host and can harbour MCR-1 (Fernandes *et al.*, 2016 ▸; Li, Yang *et al.*, 2016 ▸) alongside extended-spectrum β-lactamases such as TEM and CTX-M (Li, Xie *et al.*, 2016 ▸; Falgenhauer *et al.*, 2016 ▸; Lo *et al.*, 2014 ▸). MCR-2 (538 residues) is 81% identical to MCR-1 (Fig. 1 ▸), with 101 amino-acid substitutions (61 in the transmembrane domain and 40 in the catalytic domain) and three deletions (Met1 and Leu68 in the transmembrane domain and Gln501 in the catalytic domain; MCR-1 numbering is used throughout). Residues previously identified as essential (Glu246, His395 and the phosphorylation site Thr285) or important (Lys333, Glu468 and His478) for MCR-1 activity (Hinchliffe *et al.*, 2017 ▸) are strictly conserved in MCR-2 (red or yellow triangles, respectively, in Fig. 1 ▸), indicating a likely identical catalytic mechanism. To understand MCR diversity, we have solved the crystal structure of the MCR-2 catalytic domain (residues 217–538; MCR-2^CD^), which is 87% identical to MCR-1^CD^.

## 2. Materials and methods   

### 2.1. Macromolecule production   

To facilitate structural studies, we removed the transmembrane domain and synthesized *mcr-2* codons 217–538 (Eurofins), and subcloned them into pOPIN-F (Berrow *et al.*, 2007 ▸) using the primers in Table 1 ▸, resulting in plasmid pOPINF-MCR2^217–538^ encoding N-terminally His_6_-tagged protein (Table 1 ▸). The protein was purified as for MCR-1^CD^ (Hinchliffe *et al.*, 2017 ▸). Briefly, *E. coli* SoluBL21 cells bearing pOPINF-MCR2^217–538^ were induced at 18°C with IPTG overnight and the protein was purified using Ni–NTA affinity chromatography. The buffers contained 100 µ*M* ZnCl_2_ throughout, and the tag was removed by 3C protease cleavage and captured on Ni–NTA resin. Protein was loaded onto a Superdex 75 size-exclusion column equilibrated in 50 m*M* HEPES pH 7.5, 150 m*M* NaCl, 100 µ*M* ZnCl_2_. As for MCR-1^CD^ (Hinchliffe *et al.*, 2017 ▸; Ma *et al.*, 2016 ▸), MCR-2^CD^ eluted from the Superdex 75 column as a monomer. Peak fractions were concentrated to 15 mg ml^−1^ by centrifugation.

### 2.2. Crystallization   

Crystallization screens were conducted in MRC 2-drop 96-well sitting-drop plates using commercially available sparse-matrix screens (JCSG-*plus*, ProPlex, Structure Screen 1 + 2, Morpheus and PACT Premier from Molecular Dimensions). Crystals were obtained by mixing 0.4 µl protein solution (15 mg ml^−1^) with 0.2 µl reservoir solution (0.1 *M* KSCN, 30% PEG 2000 MME) and equilibrating against 50 µl reservoir solution (Table 2 ▸), were harvested in reservoir plus 25% glycerol and were flash-cooled in liquid nitrogen.

### 2.3. Data collection and processing   

X-ray data (Table 3 ▸) were collected at 100 K on beamline I04 at Diamond Light Source (DLS), UK, integrated in *DIALS* (Waterman *et al.*, 2016 ▸) and scaled using *AIMLESS* (Evans & Murshudov, 2013 ▸) in the *CCP*4 suite (Winn *et al.*, 2011 ▸).

### 2.4. Structure solution and refinement   

Crystallographic phases were solved using *Phaser* (McCoy *et al.*, 2007 ▸) with MCR-1^CD^ (PDB entry 5lrn) as the starting model. Variable amino acids were altered to the MCR-2 sequence and the model was completed by iterative rounds of manual model building and refinement in *Coot* (Emsley *et al.*, 2010 ▸) and *PHENIX* (Adams *et al.*, 2010 ▸). *B* factors were refined anisotropically, except for H atoms and water molecules, which were refined isotropically. Structure validation was assisted by *MolProbity* (Chen *et al.*, 2010 ▸) and *PHENIX*. Details of the refinement statistics are shown in Table 4 ▸. Atomic coordinates and structure factors have been deposited in the Protein Data Bank (PDB entry 5mx9).

## 3. Results and discussion   

**Figure 2:**
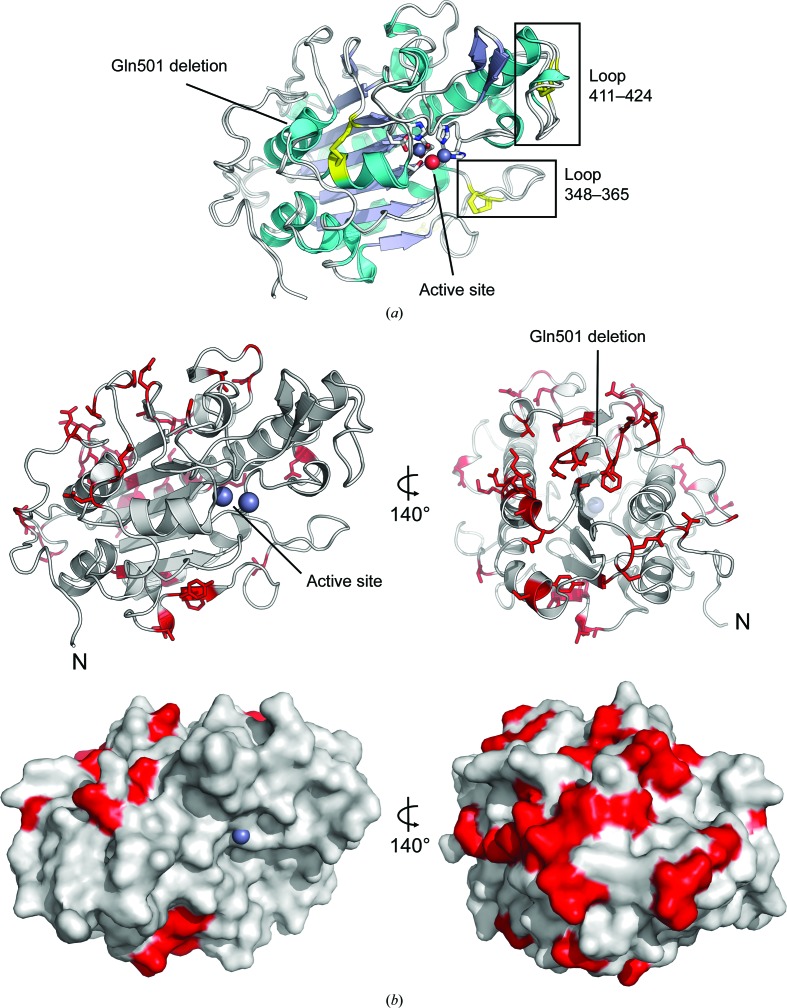
Structural comparisons of MCR-1 and MCR-2. (*a*) Alignment of MCR-1^5LRN^ and MCR-2^CD^, both coloured by secondary structure (loops are in grey, α-­helices in cyan and β-sheets in blue). The MCR-2 active site is shown (Zn spheres are in grey, waters are shown as red spheres and zinc-coordinating residues are shown as sticks). (*b*) Positions of variable amino acids (red) in MCR-2. The di-zinc (grey spheres) active site (labelled) is located on the putative membrane-proximal face. Top: two views of MCR-2 rotated 140°, with variable amino acids shown as sticks. Bottom: MCR-2 surface view, with orientations as in (*a*).

The overall MCR-2^CD^ fold contains three disulfide bonds and is essentially identical to that of MCR-1^5LRN^ (root-mean-square deviation of 0.54 Å over 314 C^α^ atoms calculated using *PDB­eFold*; Krissinel & Henrick, 2004 ▸; Fig. 2 ▸ *a*). A single residue (Ser330) is a Ramachandran plot outlier, with φ and ψ values of −165.9 and −82.4°, respectively. This residue is sterically strained by forming a hydrogen bond to Asn329, and is also a Ramachadran plot outlier in all other MCR-1 structures. Solvent-accessible loops are largely unperturbed, although loop 411–424 shifts ∼4 Å between MCR-1^5GRR^ and MCR-2^CD^. Based on comparison with the more distantly related phosphoethanolamine transferases LptA (Wanty *et al.*, 2013 ▸) and EptC (Fage *et al.*, 2014 ▸), which have 36 and 35% sequence identity to MCR-2, respectively, loop 348–365 of MCR-2 (Fig. 2 ▸ *a*) was suggested to be flexible and in an open conformation for substrate entry (Ma *et al.*, 2016 ▸). However, it makes significant crystal contacts and is in similar conformations (maximum movement of 1.5 Å) in all physiologically relevant MCR structures, with low *B* factors in MCR-2^CD^ (11.6 Å^2^). Differences in this loop compared with LptA and EptC are likely to be because the loop is longer in MCR proteins (18 residues compared with 15 and four for LptA and EptC, respectively), and may not be relevant for substrate entry. The variable amino acids of MCR-2 compared with MCR-1 are distant from both the active site and the relatively flat, proposed membrane-proximal face of the molecule (Fig. 2 ▸ *b*). Indeed, most are located on the surface, likely facing the periplasm, with the exception of Ser459Ala on the central β-sheet. The Gln501 deletion results in a periplasmic exposed loop (Fig. 2 ▸ *b*), rather than helical turn as in MCR-1, but is also distant from the active site. The effect of these variable amino acids on the activity is therefore likely to be minimal. However, this requires *in vitro* verification once both recombinant full-length enzyme is available and an assay with a suitable substrate has been developed.

**Figure 3:**
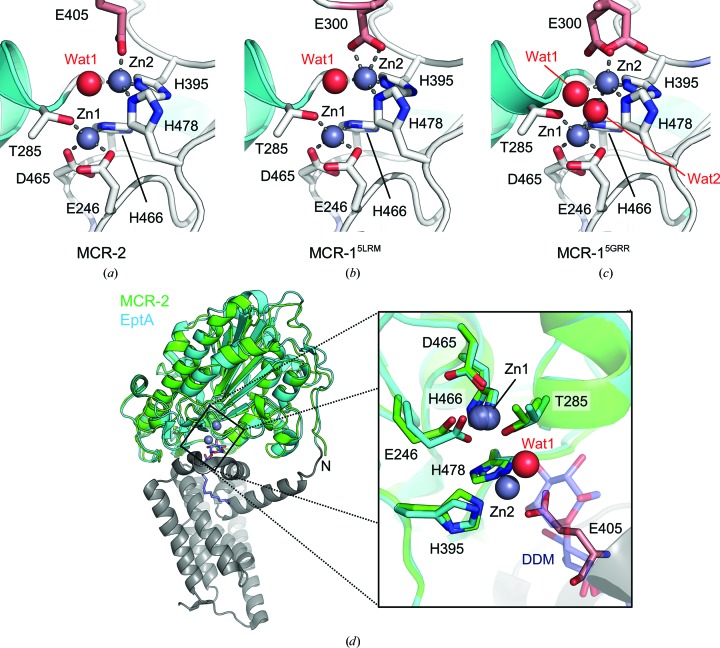
Comparison of MCR-2 with di-zinc MCR-1 and full-length EptA. (*a*) A close-up view of the MCR-2 di-zinc active site. Representations are as in Fig. 2 ▸. Residues from symmetry-related molecules are coloured light red. (*b*) MCR-1^5LRM^ active site. (*c*) MCR-1^5GRR^ active site. (*d*) Superposition of the MCR-­2 catalytic domain with full-length EptA. The catalytic domains of MCR-2 (green) and EptA (cyan; PDB entry 5fgn; Anandan *et al.*, 2017 ▸) are superposed. The membrane domain of EptA is coloured grey; zinc ions are shown as grey spheres and bound DDM as purple sticks. Left: overall view with the N-terminus of MCR-2 labelled. Right: close-up of the active site with zinc-coordinating residues shown as sticks and the Zn2-coordinating water shown as a red sphere. MCR-2 Glu405 from a symmetry-related molecule is coloured pink.

MCR-2^CD^ contains a nonphosphorylated Thr285 and clear density indicating two metal ions in the active site, modelled as zinc based on the presence of 100 µ*M* zinc in the purification buffers and homology to MCR-1, in which zinc was identified based on X-ray fluorescence scans and density functional theory calculations (Fig. 3 ▸ *a*). As for Zn1 in MCR-1, Zn1 in MCR-2 is coordinated by Glu246, Thr285 and Asp465 (all with a coordination distance of 1.92 Å) and His466 (2.04 Å) in a tetrahedral geometry (Supplementary Table S1). Although the Zn1 coordination distances are shorter in MCR-2 compared with MCR-1 (Supplementary Table S1; Hinchliffe *et al.*, 2017 ▸), there are no other structural differences around the Zn1 site, further underlying the importance of Zn1 to enzyme function. Similar to as in MCR-1^5LRM^ (Fig. 3 ▸ *b*), Zn2 in MCR-2 forms a tetrahedral geometry and is coordinated by His395, His478, a tightly bound water molecule (*B* factor of 11.97 Å^2^) and Glu405 from a symmetry-related molecule. In MCR-1^5LRM^ this latter coordination is instead provided by Glu300 owing to substantially different crystal packing. This further highlights the likely lack of physiological relevance of the MCR dimer (Ma *et al.*, 2016 ▸; Hinchliffe *et al.*, 2017 ▸) but suggests that the second zinc site can tolerate varying co­ordinating ligands. The Zn2 site is unoccupied in the two nonphysiological, high zinc-content MCR-1 structures reported previously (MCR-1^5GOV^ and MCR-1^5K4P^). MCR-1^5GRR^ is similar but contains an additional water molecule bridging Zn1 and Zn2 (Wat2; Fig. 3 ▸ *c*). However, this water molecule has a high *B* factor (51.5 Å^2^), relatively low occupancy (0.8) and little corresponding electron density and is not in any other MCR structure, suggesting that it is nonphysiological and should not be considered in mechanistic discussions.

Superposition with the full-length MCR homologue EptA (root-mean-square deviation of 0.660 Å over 213 C^α^ catalytic domain residues; Fig. 3 ▸ *d*, left) reveals close structural similarity between the two, as noted previously on comparison of the catalytic domains MCR-1^CD^ and EptA^CD^ (Hinchliffe *et al.*, 2017 ▸; Wanty *et al.*, 2013 ▸). Indeed, despite differences in zinc occupancy (two zincs in MCR-2 and one in EptA), and the presence of DDM in EptA, the conserved active-site residues adopt similar conformations, except for small differences of the conserved His395 and His478 residues that coordinate Zn2 in MCR-2^CD^ (Fig. 3 ▸ *d*, right). In EptA, His478 coordinates a DDM molecule, suggesting a possible role for these residues in positioning the substrate rather than binding a second zinc ion. However, it cannot be ruled out that physiological substrates (*i.e.* lipid A or phosphatidylethanolamine) could replace the Glu300/Glu405–Zn2 coordination in recruiting a second zinc ion during the mechanism (Wanty *et al.*, 2013 ▸).

The physiological relevance of the second zinc site has yet to be established, although it has now been observed in three MCR crystal structures. Our density functional theory calculations (Hinchliffe *et al.*, 2017 ▸) suggest a two-zinc mechanism to be feasible for MCR-1, although a one-zinc mechanism was tentatively more favourable. Resolving this issue will require accurate and detailed mechanistic and computational studies of phosphoethanolamine transfer by the MCR family of enzymes, the latter of which will be greatly facilitated by the exceptionally high resolution of the current structure. The MCR-2^CD^ structure also indicates that amino-acid mutations on the periplasmic facing surface of MCR-1 are well tolerated. This, together with the wide geographic distribution of MCR-1 and the intense current research in this area, makes it likely that further clinical MCR variants will be identified in due course. Thus, achieving full understanding of mobile colistin resistance will require consideration, including structural and biochemical characterization, of family members beyond MCR-1. The current structure represents a first step towards this goal.

## Supplementary Material

PDB reference: MCR-2 catalytic domain, 5mx9

Supplementary Table S1. DOI: 10.1107/S2053230X17009669/no5117sup1.pdf

## Figures and Tables

**Table 1:** Macromolecule-production information Lower-case letters in the primers indicate overlap with pOPIN-F for In-Fusion cloning. The His_6_ tag is in shown in italics and the 3C protease cleavage site is underlined in the construct sequence.

Source organism	*E. coli*
DNA source	Synthetic codon-optimized gene
Forward primer	aagttctgtttcagggcccgACCATCTATCACGCCAAAGATGCG
Reverse primer	atggtctagaaagctttaCTGGATAAACGCAGCACGGTC
Cloning vector	pEX-A2
Expression vector	pOPIN-F
Expression host	*E. coli* SoluBL21
Complete amino-acid sequence of the construct produced	MA*HHHHHH* SSGLEVL GPTIYHAKDAVQTTKPSERKPRLVVFVVGETARADHVQFNGYGRETFPQLAKVDGLANFSQVTSCGTSTAYSVPCMFSYLGQDDYDVDTAKYQENVLDTLDRLGVGILWRDNNSDSKGVMDKLPATQYFDYKSATNNTICNTNPYNECRDVGMLVGLDDYVSANNGKDMLIMLHQMGNHGPAYFKRYDEQFAKFTPVCEGNELAKCEHQSLINAYDNALLATDDFIAKSIDWLKTHEANYDVAMLYVSDHGESLGENGVYLHGMPNAFAPKEQRAVPAFFWSNNTTFKPTASDTVLTHDAITPTLLKLFDVTAGKVKDRAAFIQ

**Table 2:** Crystallization

Method	Sitting-drop vapour diffusion
Plate type	MRC 2-drop 96-well
Temperature (K)	291
Protein concentration (mg ml^−1^)	15
Buffer composition of protein solution	50 m*M* HEPES pH 7.5, 150 m*M* NaCl, 100 µ*M* ZnCl_2_
Composition of reservoir solution	0.1 *M* KSCN, 30% PEG 2000 MME
Volume and ratio of drop	0.4 µl protein solution, 0.2 µl reservoir solution
Volume of reservoir (µl)	50

**Table 3:** Data collection and processing Values in parentheses are for the outer shell.

Diffraction source	Beamline I04, DLS
Wavelength (Å)	0.97949
Temperature (K)	100
Detector	PILATUS 6M-F
Crystal-to-detector distance (mm)	187.63
Rotation range per image (°)	0.2
Total rotation range (°)	360
Exposure time per image (s)	0.1
Space group	*P*2_1_2_1_2_1_
*a*, *b*, *c* (Å)	44.82, 53.31, 117.51
α, β, γ (°)	90, 90, 90
Mosaicity (°)	0.133
Resolution range (Å)	58.75–1.12 (1.14–1.12)
Total No. of reflections	1276800 (31833)
No. of unique reflections	108781 (5133)
Completeness (%)	99.8 (96.8)
Multiplicity	11.7 (6.2)
〈*I*/σ(*I*)〉	20.4 (3.4)
*R*_r.i.m._	0.017 (0.192)
CC_1/2_	1.000 (0.879)
Overall *B* factor from Wilson plot (Å^2^)	8.361

**Table 4:** Structure refinement Values in parentheses are for the outer shell.

Resolution range (Å)	48.548–1.120
Completeness (%)	99.8
No. of reflections, working set	108679
No. of reflections, test set	5480
Final *R*_cryst_	0.1334
Final *R*_free_	0.1453
No. of non-H atoms	
Protein	2565
Zinc	2
Solvent	437
Total	3004
R.m.s. deviations	
Bonds (Å)	0.008
Angles (°)	1.382
Average *B* factors (Å^2^)	
Protein	11.57
Zinc	10.48
Solvent	25.79
Ramachandran plot	
Favoured regions (%)	97.86
Additionally allowed (%)	1.83
Outliers (%)	0.31

## References

[bb1] Adams, P. D. *et al.* (2010). *Acta Cryst.* D**66**, 213–221.

[bb2] Anandan, A., Evans, G. L., Condic-Jurkic, K., O’Mara, M. L., John, C. M., Phillips, N. J., Jarvis, G. A., Wills, S. S., Stubbs, K. A., Moraes, I., Kahler, C. M. & Vrielink, A. (2017). *Proc. Natl Acad. Sci. USA*, **114**, 2218–2223. 10.1073/pnas.1612927114 PMC5338521 28193899

[bb3] Berrow, N. S., Alderton, D., Sainsbury, S., Nettleship, J., Assenberg, R., Rahman, N., Stuart, D. I. & Owens, R. J. (2007). *Nucleic Acids Res.* **35**, e45. 10.1093/nar/gkm047 PMC1874605 17317681

[bb4] Biswas, S., Brunel, J.-M., Dubus, J.-C., Reynaud-Gaubert, M. & Rolain, J.-M. (2012). *Expert Rev. Anti Infect. Ther.* **10**, 917–934. 10.1586/eri.12.78 23030331

[bb5] Chen, V. B., Arendall, W. B., Headd, J. J., Keedy, D. A., Immormino, R. M., Kapral, G. J., Murray, L. W., Richardson, J. S. & Richardson, D. C. (2010). *Acta Cryst.* D**66**, 12–21. 10.1107/S0907444909042073 PMC2803126 20057044

[bb6] Clausell, A., Garcia-Subirats, M., Pujol, M., Busquets, M. A., Rabanal, F. & Cajal, Y. (2007). *J. Phys. Chem. B*, **111**, 551–563. 10.1021/jp064757+ 17228913

[bb7] Di Pilato, V., Arena, F., Tascini, C., Cannatelli, A., Henrici De Angelis, L., Fortunato, S., Giani, T., Menichetti, F. & Rossolini, G. M. (2016). *Antimicrob. Agents Chemother.* **60**, 5612–5615. 10.1128/AAC.01075-16 PMC4997870 27401575

[bb8] Emsley, P., Lohkamp, B., Scott, W. G. & Cowtan, K. (2010). *Acta Cryst.* D**66**, 486–501. 10.1107/S0907444910007493 PMC2852313 20383002

[bb9] Evans, P. R. & Murshudov, G. N. (2013). *Acta Cryst.* D**69**, 1204–1214. 10.1107/S0907444913000061 PMC3689523 23793146

[bb10] Fage, C. D., Brown, D. B., Boll, J. M., Keatinge-Clay, A. T. & Trent, M. S. (2014). *Acta Cryst.* D**70**, 2730–2739. 10.1107/S1399004714017623 PMC4188012 25286856

[bb11] Falgenhauer, L., Waezsada, S. E., Yao, Y., Imirzalioglu, C., Käsbohrer, A., Roesler, U., Michael, G. B., Schwarz, S., Werner, G., Kreienbrock, L. & Chakraborty, T. (2016). *Lancet Infect. Dis.* **16**, 282–283. 10.1016/S1473-3099(16)00009-8 26774242

[bb12] Fernandes, M. R., McCulloch, J. A., Vianello, M. A., Moura, Q., Perez-Chaparro, P. J., Esposito, F., Sartori, L., Dropa, M., Matte, M. H., Lira, D. P., Mamizuka, E. M. & Lincopan, N. (2016). *Antimicrob. Agents Chemother.* **60**, 6415–6417. 10.1128/AAC.01325-16 PMC5038249 27503650

[bb13] Haenni, M., Poirel, L., Kieffer, N., Chatre, P., Saras, E., Metayer, V., Dumoulin, R., Nordmann, P. & Madec, J.-Y. (2016). *Lancet Infect. Dis.* **16**, 281–282. 10.1016/S1473-3099(16)00007-4 26774244

[bb14] Hinchliffe, P. *et al.* (2017). *Sci. Rep.* **7**, 39392. 10.1038/srep39392 PMC5216409 28059088

[bb15] Hu, M., Guo, J., Cheng, Q., Yang, Z., Chan, E. W. C., Chen, S. & Hao, Q. (2016). *Sci. Rep.* **6**, 38793. 10.1038/srep38793 PMC5153839 27958270

[bb16] Karaiskos, I., Souli, M., Galani, I. & Giamarellou, H. (2017). *Expert Opin. Drug Metab. Toxicol.* **13**, 59–71. 10.1080/17425255.2017.1230200 27573251

[bb17] Krissinel, E. & Henrick, K. (2004). *Acta Cryst.* D**60**, 2256–2268. 10.1107/S0907444904026460 15572779

[bb19] Li, A., Yang, Y., Miao, M., Chavda, K. D., Mediavilla, J. R., Xie, X., Feng, P., Tang, Y.-W., Kreiswirth, B. N., Chen, L. & Du, H. (2016). *Antimicrob. Agents Chemother.* **60**, 4351–4354. 10.1128/AAC.00550-16 PMC4914624 27090180

[bb18] Li, R., Xie, M., Zhang, J., Yang, Z., Liu, L., Liu, X., Zheng, Z., Chan, E. W.-C. & Chen, S. (2016). *J. Antimicrob Chemother.* **72**, 393–401. 10.1093/jac/dkw411 28073961

[bb20] Liu, Y.-Y. *et al.* (2016). *Lancet Infect. Dis.* **16**, 161–168.

[bb21] Lo, W.-U., Chow, K.-H., Law, P. Y., Ng, K.-Y., Cheung, Y.-Y., Lai, E. L. & Ho, P.-L. (2014). *J. Med. Microbiol.* **63**, 835–840. 10.1099/jmm.0.074021-0 24595536

[bb22] Ma, G., Zhu, Y., Yu, Z., Ahmad, A. & Zhang, H. (2016). *Sci. Rep.* **6**, 39540. 10.1038/srep39540 PMC5175174 28000749

[bb23] McCoy, A. J., Grosse-Kunstleve, R. W., Adams, P. D., Winn, M. D., Storoni, L. C. & Read, R. J. (2007). *J. Appl. Cryst.* **40**, 658–674. 10.1107/S0021889807021206 PMC2483472 19461840

[bb24] Mediavilla, J. R., Patrawalla, A., Chen, L., Chavda, K. D., Mathema, B., Vinnard, C., Dever, L. L. & Kreiswirth, B. N. (2016). *MBio*, **7**, e01191-16. 10.1128/mBio.01191-16 PMC4999550 27578755

[bb25] Stojanoski, V., Sankaran, B., Prasad, B. V., Poirel, L., Nordmann, P. & Palzkill, T. (2016). *BMC Biol.* **14**, 81. 10.1186/s12915-016-0303-0 PMC5031297 27655155

[bb26] Wanty, C., Anandan, A., Piek, S., Walshe, J., Ganguly, J., Carlson, R. W., Stubbs, K. A., Kahler, C. M. & Vrielink, A. (2013). *J. Mol. Biol.* **425**, 3389–3402. 10.1016/j.jmb.2013.06.029 23810904

[bb27] Waterman, D. G., Winter, G., Gildea, R. J., Parkhurst, J. M., Brewster, A. S., Sauter, N. K. & Evans, G. (2016). *Acta Cryst.* D**72**, 558–575. 10.1107/S2059798316002187 PMC4822564 27050135

[bb28] Wiese, A., Gutsmann, T. & Seydel, U. (2003). *J. Endotoxin Res.* **9**, 67–84. 10.1179/096805103125001441 12803879

[bb29] Winn, M. D. *et al.* (2011). *Acta Cryst.* D**67**, 235–242.

[bb30] Xavier, B. B., Lammens, C., Ruhal, R., Kumar-Singh, S., Butaye, P., Goossens, H. & Malhotra-Kumar, S. (2016). *Euro Surveill.* **21**, https://doi.org/10.2807/1560-7917.ES.2016.21.27.30280. 10.2807/1560-7917.ES.2016.21.27.30280 27416987

