# Supplementary material for: 1.12 Å resolution crystal structure of the catalytic domain of the plasmid-mediated colistin resistance determinant MCR-2
Source: Acta Crystallogr F Struct Biol Commun. 2017 Jul 26;73(Pt 8):443–9. doi: 10.1107/S2053230X17009669 (PMC5544000; doi:10.1107/S2053230X17009669)
Supplement: Supplementary file 1 [file f-73-00443-sup1.pdf]

Acta Cryst F

STRUCTURAL BIOLOGY
COMMUNICATIONS

Volume 73 (2017)

Supporting information for article:

1.12 Å resolution crystal structure of the catalytic domain of the plasmid-mediated colistin resistance determinant MCR-2

Katie Coates, Timothy R. Walsh, James Spencer and Philip Hinchliffe

Supplementary Table S1. MCR-1 and MCR-2 zinc-coordination distances in Å

		MCR-2		MCR-1[5LRM]	
**residue**	**atom**	**Zn1**	**Zn2**	**Zn1**	**Zn2**
E405/E300 (symmetry)	Oε2	-	1.95	-	2.12
	Oε1	-	2.86	-	2.31
Wat1	O	-	1.91	-	1.91
His395	Nε2	-	2.00	-	1.87
His478	Nε2	-	1.93	-	2.04
Glu246	Oε2	1.92	-	2.04	-
Asp465	Oδ1	1.92	-	1.99	-
Thr285	Oγ1	1.92	-	2.10	-
His466	Nε2	2.04	-	2.10	-
